# A Modified Communication and Optimal Resolution Program for Intersystem Medical Error Discovery: Protocol for an Implementation Study

**DOI:** 10.2196/13396

**Published:** 2019-07-02

**Authors:** Lesly Dossett, Jacquelyn Miller, Reshma Jagsi, Anne Sales, Michael D Fetters, Richard C Boothman, Justin B Dimick

**Affiliations:** 1 Center for Health Outcomes and Policy, Institute for Health Policy and Innovation Department of Surgery University of Michigan Ann Arbor, MI United States; 2 Center for Bioethics and Social Sciences Medicine University of Michigan Ann Arbor, MI United States; 3 Department of Radiation Oncology University of Michigan Ann Arbor, MI United States; 4 Ann Arbor VA Center for Clinical Management Research Ann Arbor, MI United States; 5 Mixed Methods Research and Scholarship Program Department of Family Medicine Ann Arbor, MI United States; 6 Department of Surgery University of Michigan Ann Arbor, MI United States

**Keywords:** medical error, patient safety, disclosure, communication, feedback, ethics, implementation science

## Abstract

**Background:**

Preventable medical errors represent a major public health problem. To prevent future errors, improve disclosure, and mitigate malpractice risks, organizations have adopted strategies for transparent communication and emphasized quality improvement through peer review. These principles are incorporated into the Agency for Healthcare Research and Quality (AHRQ) Communication and Optimal Resolution (CANDOR) Toolkit, which facilitates (1) transparent communication, (2) error prevention, and (3) achieving optimal resolution with patients and families; however, how medical errors should be addressed when they are discovered between systems—intersystem medical error discovery (IMED)—remains unclear. Without mechanisms for disclosure and feedback on the part of the discovering provider, uncertainty remains as to the extent to which IMED is communicated with patients or responsible providers. Furthermore, known barriers to disclosure and reporting one’s own error may not be relevant or may be replaced by other unknown barriers when considering scenarios of IMED.

**Objective:**

This study aims to develop and test implementation of a modified CANDOR process for application to IMED scenarios.

**Methods:**

We plan a series of studies following an implementation framework. First, we plan a participatory, consensus-building stakeholder panel process to develop the modified CANDOR process. We will then conduct a robust preimplementation analysis to identify determinants of implementation of the modified process. Using the Consolidated Framework for Implementation Research as a theoretical framework, we will assess organizational readiness by key informant interviews and individual-level behaviors by a survey. Findings from this analysis will inform the implementation toolkit that will be developed and pilot-tested at 2 cancer centers, sites where IMED is hypothesized to occur more frequently than other settings. We will measure 5 implementation outcomes (acceptability, appropriateness, reach, adoption, and feasibility) using a combination of key informant interviews and surveys over the pre- and postimplementation phases.

**Results:**

This protocol was funded in August 2018 with support from the AHRQ. The University of Michigan Medical School Institutional Review Board has reviewed and approved the scope of activities described. As of April 2019, step 1 of aim 1 is underway, and aim 1 is projected to be completed by April 2020. Data collection is projected to begin in January 2020 for aim 2 and in August 2020 for aim 3.

**Conclusions:**

Providing a communication and resolution strategy applicable to IMED scenarios will help address the current blind spot in the patient safety movement. This work will provide important insights into the potential utility of an implementation toolkit to improve transparent communication and optimal resolution of IMED scenarios. The natural progression of this work will be to test the toolkit more broadly, understand the feasibility and barriers of implementation on a broader scale, and pilot the implementation in new organizations.

**International Registered Report Identifier (IRRID):**

PRR1-10.2196/13396

## Introduction

### Background

Preventable medical errors represent a major public health problem. To prevent future errors, improve disclosure, and mitigate malpractice risks, organizations have adopted strategies for early transparent communication and emphasized quality improvement through peer review [1-4]. The Agency for Healthcare Research and Quality (AHRQ) Communication and Optimal Resolution (CANDOR) process integrates these practices in a comprehensive response to medical errors, which aims to improve safety and optimize resolution for patients, providers, and health systems [4,5]. Institutions and practitioners can use the CANDOR process to respond in a timely, thorough, and just manner to unexpected events that might result in harm to patients. The major tenets of the CANDOR process are (1) transparent communication with patients and families, (2) incident reporting and safety program review, and (3) risk management and resolution programs.

However, what physicians should do after identifying an error from another system—intersystem medical error discovery (IMED)—is less clear. The literature [6-8], anecdotal experience [9], and our previous work [10,11] draw attention to scenarios where providers discover errors originating from other systems. These errors may be unknown to the patient and/or responsible provider. Although the CANDOR principles of transparent communication and optimal resolution of other physicians’ errors remain possible when the physicians practice in the same system (*within system*), it is uncertain how CANDOR principles are best applied to IMED scenarios. Through the CANDOR Toolkit, AHRQ provides clear guidance on how to implement the CANDOR process within an institution, including guidelines for preimplementation assessments, gap analyses, and obtaining organizational buy-in. It includes a CANDOR event checklist and best practices for event reporting, investigation, and resolution [5]. In contrast, there are no guidelines or mechanisms for reporting and investigating errors that are discovered between systems or for providing disclosure or resolution to the patient in such scenarios.

### Objective

Identification of errors between systems is particularly relevant to complex oncologic care where patients often interact with multiple systems and where specialists are highly dependent on external referrals. Solutions to IMED developed in this challenging context should be adaptable to similarly complex settings. Our preliminary work suggests cancer specialists regularly encounter IMED scenarios but lack consensus on whether or how to communicate about these errors to patients and responsible providers. Specialists struggled to provide disclosure to patients or meaningful feedback to responsible providers. Barriers to transparent communication included concern for medicolegal implications, disruptions to referral relationships, concern for the profession, and general discomfort with giving negative feedback to other physicians [10,11].

Without clear expectations or mechanisms for disclosure and feedback on the part of the discovering provider, it is uncertain how best to communicate about errors discovered between systems. Furthermore, known barriers to disclosure and reporting of one’s own error may not be relevant or may be replaced by other unknown barriers when considering IMED. The objective of this study is to provide a communication and resolution strategy applicable to IMED scenarios to help address this current blind spot in the patient safety and quality improvement movement.

## Methods

### Overall Design

**Figure 1:**
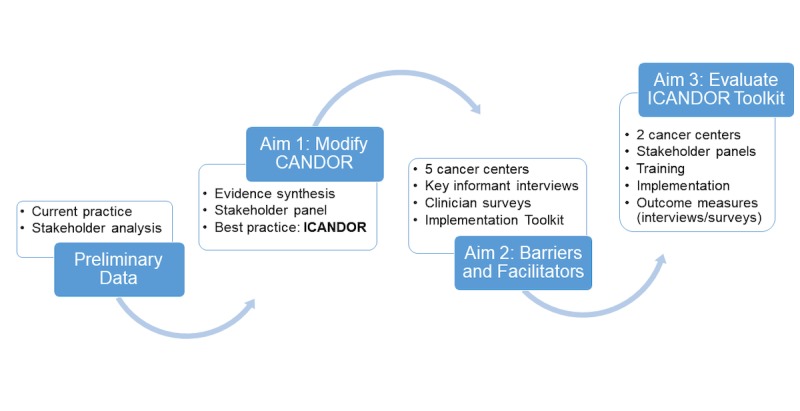
Overview of studies and data flow. CANDOR: Communication and Optimal Resolution; ICANDOR: Intersystem Communication and Optimal Resolution.

We plan a series of studies following an implementation framework [12] where we use best (aim 1) and current practice (preliminary data) to identify a practice gap. We will then conduct a robust preimplementation analysis to identify barriers and facilitators to implementation using the Consolidated Framework for Implementation Research (CFIR) [13,14] supplemented by the Theoretical Domains Framework (TDF) [15] (aim 2). Findings from aim 2 will inform the implementation toolkit that will be developed and pilot-tested in aim 3 (Figure 1).

### Aim 1: Modify the Communication and Optimal Resolution Process for Application to Errors Discovered Between Systems

#### Introduction

The tenets of the CANDOR process are (1) transparent communication with patients, (2) incident reporting and safety program review, and (3) risk management and resolution. These tenets are accomplished through 5 process components: (1) event identification, (2) system activation, (3) response and disclosure, (4) event investigation, and (5) resolution. CANDOR is designed to apply *within* systems; no current process addresses communication or resolution for IMED scenarios.

#### Research Design

On the basis of a comprehensive stakeholder analysis and participatory, consensus-building stakeholder panel process [16-18], we will modify the CANDOR process for application to IMED scenarios. Experts will be recruited nationwide through professional contacts and will include leaders in clinical care, patient safety, bioethics, law, risk management, and hospital administration.

##### Step 1: Evidence Synthesis

Sources of data will include a scoping review of published ethics codes, a narrative review of legal case law relevant to feedback and reporting (completed), and a systematic review of empirical quantitative and qualitative data using previously published methodology for mixed methods meta-synthesis [19]. For the empirical qualitative data, we will use the Grading Recommendations Assessment, Development and Evaluation-Confidence in Evidence from Reviews of Qualitative Research methods for grading the evidence [20]. These reviews will collectively represent the available evidence for consideration by the stakeholder panel.

##### Step 2: Independent Review by Stakeholder Panel

We will create 8 to 10 IMED scenarios to which the evidence from step 1 may apply. Panelists will be provided with the scenarios as well as the evidence synthesis as a written report. They will be asked to propose modifications to the CANDOR process based on the evidence synthesis and their expert judgment. Participants will respond anonymously.

##### Step 3: Face-to-Face Meetings of Stakeholder Panel—Preliminary Proposals

The study team will compile the responses, and the panelists will then be brought together in a face-to-face meeting (videoconference if necessary). The aggregate proposals will be presented for discussion. During the face-to-face meeting, we will utilize nominal group technique to encourage contributions from all stakeholders and to prioritize recommendations. Through this technique, each participant will have opportunities to share their priority proposals with the group in turn. A facilitator will record and further consolidate proposals as needed. Thereafter, the group will discuss each proposal in turn and further prioritize them using the multivoting procedure. Discussions will be audio-recorded for further analysis.

##### Step 4: Summary of Recommendations by Research Team

The research team will then summarize the written and audio-recorded recommendations and deliver them to the stakeholder panel in a written report. Issues of disagreement and areas requiring further elaboration will be highlighted in the report as specific questions. The stakeholders will again be asked to propose answers to the specific questions as well as modifications or revisions to the recommendations generally. Stakeholder responses will be received by email and anonymized.

##### Step 5: Face-to-Face Meeting of Stakeholder Panel—Iterative Review and Revisions

The study team will compile the responses, and the panelists will be brought together for a second face-to-face meeting. An iterative facilitated process will follow, through which panelists will have an opportunity to provide feedback on the draft recommendations and approve the final recommendation.

##### Step 6: Final Recommendation Prepared and Disseminated by the Research Team

**Figure 2:**
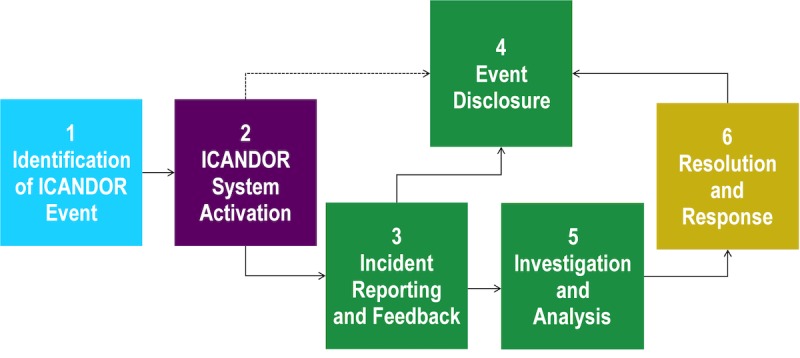
Possible modified ICANDOR process resulting from Aim 1. ICANDOR: Intersystem Communication and Optimal Resolution.

The research team will then provide final recommendations of the stakeholder panel in a published report. The expected outcome from aim 1 will be a modified process for the transparent communication and optimal resolution of errors identified between systems—the intersystem CANDOR process (ICANDOR) based on a comprehensive stakeholder analysis. The modified process (Figure 2, adapted from the CANDOR Toolkit [5]) will describe ICANDOR system activation, provide recommendations for feedback and/or reporting, and establish guidelines for disclosure in these scenarios.

### Aim 2: Understand the Barriers and Facilitators to Implementation of the Intersystem Communication and Optimal Resolution Process

#### Introduction

Although AHRQ provides implementation guidance for adoption of the CANDOR process, our preliminary data suggest that the barriers to responding to errors discovered between systems are different from those encountered within a system [10,11]. In this case, the implementation strategy for the CANDOR process (the CANDOR Toolkit) may be ineffective. For example, AHRQ recommends building a business case for CANDOR given the evidence that CANDOR may reduce medicolegal claims (*a facilitator of implementation*) [21-23]. Conversely, specialists express concern that disclosing another physician’s error may negatively impact future referrals, thereby providing a business disincentive to ICANDOR (*a barrier to implementation*) [10]. Our goal is to develop an implementation toolkit that includes key information about the implementation constructs most salient to ICANDOR dissemination and implementation and strategies for effective ICANDOR implementation.

#### Research Design

We will conduct a robust preimplementation assessment to understand organizational and individual barriers and facilitators to implementing ICANDOR. We will assess organizational readiness and culture by key informant interviews and individual-level behaviors by a cancer specialist survey. We will use the CFIR [13,14] to guide data collection and analysis, supplemented by the TDF [15] for the individual-level analysis [24].

##### Theoretical Frameworks and Instruments

CFIR is a meta-theoretical framework that comprises 39 constructs across 5 domains consolidated from published implementation theories to systematically assess contextual factors influencing practice change. Domains include intervention characteristics, outer setting (eg, external policies and incentives), inner setting (eg, implementation team communication), individual characteristics, and implementation process [13]. CFIR was selected because it provides a pragmatic, consistent typology applicable across multiple implementation contexts. Because CFIR focuses on organizational characteristics, we will supplement the survey with constructs from TDF to enable a thorough evaluation of individual behavior change constructs. TDF was developed for implementation research to identify influences on health professional behavior; it consolidates 33 theories of behavior change into 14 domains [15,25]. On the basis of our study and previous studies by others on error resolution [10,11,26], we will select the CFIR and TDF constructs most likely to be the potential determinants of implementation or to have sufficient variation across organizations.

##### Setting

**Figure 3:**
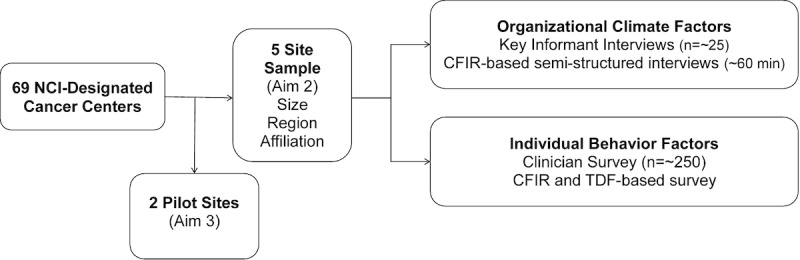
Overview of sampling strategy and study design for Aim 2. CFIR: Consolidated Framework for Implementation Research; NCI: National Cancer Institute; TDF: Theoretical Domains Framework.

We will purposively sample 5 of the 69 NCI-designated cancer centers in the United States (excluding the centers selected for pilot testing in aim 3) to maximize diversity in site characteristics (eg, size, geographic region, and affiliation with a university medical center; Figure 3). We have chosen cancer centers as the site of testing because identification of errors between facilities is particularly relevant to complex oncologic care. The screening, diagnosis, and multidisciplinary management of cancer requires patients to interface with multiple physicians and facility types with varying levels of integration [27]. In the cancer care environment, consequences of errors can be especially harmful, further complicating the willingness or responsibility for disclosure of the discovering provider [28,29]. Our preliminary work suggests cancer specialists lack consensus on whether or how to communicate about these errors to patients and responsible providers [10,11].

##### Key Informant Interviews

For key informant interviews, we will contact institutional risk management offices through publicly available data center websites or professional contacts. We will introduce our research and identify and recruit key hospital personnel at each site who led CANDOR process implementation, if applicable, or are involved in patient safety and error resolution. These may be risk managers, patient safety and quality improvement personnel, legal counsel, or clinical ethicists (3-5 interviews per site). We will use snowball sampling [30] supplemented with information-rich informants to ensure representation of diverse perspectives. Interviews (60 min approximately) will be recorded, transcribed, and imported into a software that supports qualitative/mixed methods analyses. We will analyze data using framework analysis in the following steps: (1) immerse in the details of each transcript, (2) use CFIR constructs as key themes, (3) code transcripts with CFIR framework to identify recurrent subthemes, (4) summarize data in a matrix, and (5) synthesize data by comparing across cases. Preliminary analysis will be performed iteratively with interviews to assess sample size for appropriate *information power* up to a total of 25 individuals. Information power is an approach to estimate maximum sample size in qualitative studies that considers salient study characteristics affecting the amount of relevant information a sample is likely to provide. This maximum sample size takes into account the focus of the study aim, the specificity of the sample to personnel with experience in the topic, our prior experience with the quality of dialogue with hospital personnel [10,11], and the theoretical framework that will structure both the interview guide and analysis [31].

##### Clinician Survey

A random sample of clinicians (50 per site; medical, radiation, and surgical oncologists) will be recruited to complete the survey at each of the 5 sites (n=250). A roster of physicians and their contact information will be obtained from Web-based *Find a Physician* registries of each cancer center. A research assistant will confirm accurate mailing addresses by phone before mailing. Physicians will be recruited by letter with an attached survey and a nonconditional cash incentive [32,33]. We will use the Dillman method of survey administration [34] to achieve optimal response rates with a goal of 65% [27,35-37]. The survey will be developed to determine barriers and facilitators to ICANDOR implementation, using CFIR constructs as a guide for selecting survey items, augmented by the TDF to fully explore individual behavior. The questionnaire will be pilot-tested for face validity, clarity, and stability over time with 15 participants representing all study sites, who will be excluded from subsequent participation. Face validity will be established by qualitative assessment via field notes with pilot respondents and other nonparticipant stakeholders. Clarity will be established by observing pilot participants completing the survey, asking them to think out loud as they are completing the survey, and identifying any items requiring further clarification. Finally, stability will be tested using test-retest methodology [38]. Likert scale responses will be dichotomized as follows: (1) likely/very likely versus not sure/unlikely/very unlikely. Summary statistics will include sample size, mean, median, SD, and range for continuous variables, and counts and percentages for categorical or ordinal variables.

##### Mixed Methods Analysis

Summary statistics for clinician surveys at each site will be imported to the qualitative data analysis software and linked to the qualitative analysis of key informant interviews at the site level (ie, each of the 5 sites will be analyzed as a case). This will enable mixed methods analysis by examining potential patterns in the data among the 5 sites.

#### Implementation Toolkit

The proposed activities will identify barriers and facilitators to implementation of the ICANDOR process across a diverse setting of cancer centers. From these data, we will generate an implementation toolkit for guiding ICANDOR implementation. We anticipate that multiple categories of implementation strategies will be necessary, and the final strategy will be a bundled approach [39]. Our proposed activities will also identify the CFIR constructs most salient to ICANDOR, providing a foundation for evaluating future implementation efforts.

### Aim 3: Evaluate the Acceptability and Feasibility of a Toolkit for Intersystem Communication and Optimal Resolution Implementation

#### Introduction

Although toolkits are effective interventions to facilitate practice change, there is a need to rigorously study the acceptability, utility, and impact of specific toolkit components before widespread implementation.

#### Research Design

**Figure 4:**
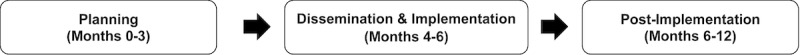
Research design and timeline for Aim 3.

We will use the implementation toolkit created in aim 2 to implement ICANDOR at 2 National Cancer Institute (NCI)–designated cancer centers with whom the study team has strong institutional ties. We will collaborate with study site stakeholders to select and refine tools and strategies from the toolkit and implement ICANDOR. We will then measure early implementation outcomes including acceptability, appropriateness, reach, adoption, and feasibility. The overall study period will be 12 months (Figure 4). Study sites will include 2 NCI-designated cancer centers with distinct representation of geographic region and affiliation.

In the planning phase, we will form stakeholder panels (eg, clinicians, legal experts, bioethicists, risk officers, and patients) at each site. We will present the ICANDOR Toolkit from aim 2 to the stakeholder panels and elicit feedback on site-specific barriers/facilitators and the toolkit strategies perceived as acceptable and useful. We will then use a rapid assessment approach [40] to balance rigor with timeliness in the analysis and synthesis of data, review key stakeholder recommendations, and specify final implementation interventions. At the end of the planning period, we will conduct training sessions among cancer specialists participating in 3 multidisciplinary tumor boards at each site (eg, Sarcoma, Colorectal, Thoracic, Gynecologic Oncology) in error identification between systems and ICANDOR (active dissemination), as well as the use of selected implementation strategies. We will initiate ICANDOR at the study sites (month 4). In months 4 to 12, we will collect data on implementation outcomes including adoption (month 4-6) and appropriateness, reach, acceptability, and feasibility (month 12).

##### Outcome Measures

We will measure 5 implementation outcomes from the Proctor et al [41] taxonomy of outcomes—acceptability, appropriateness, reach, adoption, and feasibility (Table 1). To assess initial acceptability, appropriateness, and feasibility, we will conduct key informant interviews (n=10 at each site, or until appropriate information power is achieved [31]) in the planning phase. Following the clinician training in the dissemination and implementation phase, we will conduct short posttraining surveys to measure adoption. Finally, at month 12, we will invite all cancer specialists participating in the training sessions (estimated n=100) and the error resolution staff (n=20) during the study period to complete surveys to reassess acceptability, appropriateness, and feasibility. To gain a greater understanding about the quantitative survey findings, we will then purposively sample 2 or 3 respondents within each stakeholder type at each site (n=30 total, or until appropriate information power is achieved [31]) with very high or very low scores to participate in semistructured interviews. We will also measure reach by determining the number of unique providers who report an ICANDOR event, triggering use of the implementation toolkit, during the study period.

**Table 1:** Summary of dissemination and implementation outcomes, method, and timing of measurement.

Construct	Definition	Method of measurement	Timing
Acceptability	Perception among implementation stakeholders that the toolkit is agreeable, palatable, or satisfactory	Key Informant Interviews; Clinician Survey	Planning; postimplementation
Appropriateness	Perceived fit, relevance, or compatibility of the toolkit for the particular practice setting	Key Informant Interview; Clinician Survey	Planning; postimplementation
Reach/penetration	The number of providers who report an ICANDOR^a^ event divided by the number of providers who participated in the training	Reporting Data	Postimplementation
Adoption	The intention, initial decision, or action to try or employ the ICANDOR Toolkit	Clinician Survey	Posttraining
Feasibility	The extent to which the ICANDOR Toolkit can be successfully carried out	Key Informant Interviews; Clinician Survey	Planning; postimplementation

^a^ICANDOR: Intersystem Communication and Optimal Resolution.

##### Analysis

Given this study’s focus on acceptability and feasibility, our analysis will be primarily descriptive. For analysis of the quantitative data, we will calculate descriptive and bivariate statistics on survey responses (acceptability and appropriateness). We will then generate mean acceptability and appropriateness scores for each component of the implementation toolkit. For key informant interviews, all meetings will be recorded, transcribed, and analyzed using rapid assessment. Using a joint display organized by prespecified implementation activities, we will visually merge findings from qualitative and quantitative data analysis, presenting quantitative scores with representative qualitative quotes. Monthly ICANDOR adoption rates in the 6 months after pilot implementation will be measured. We will present findings to the stakeholder panels and refine the implementation toolkit to include a detailed description of the implementation planning process, advice about toolkit use, and improved strategies and tools.

## Results

This protocol was funded in August 2018 with support from the AHRQ (see Multimedia Appendix 1). The University of Michigan Medical School Institutional Review Board has reviewed and approved the scope of activities described (study ID HUM00151593). As of April 2019, step 1 of aim 1 is underway, and aim 1 is projected to be completed by April 2020. Data collection is projected to begin in January 2020 for aim 2 and in August 2020 for aim 3.

## Discussion

Providing a communication and resolution strategy applicable to IMED would help address this current blind spot in the patient safety and quality improvement movement. The proposed work will generate a refined toolkit to guide ICANDOR dissemination and implementation more broadly, thereby improving response to errors discovered between systems. The natural progression of this work will be to test the toolkit more broadly, understand the feasibility and barriers of implementation on a broader scale, and pilot the implementation in new organizations.

This study has several potential limitations, which we have attempted to mitigate. We may find that the components of CANDOR are not easily modified to apply to ICANDOR events, or that new components need to be added. In this case, we will rely on the expertise of the panelists to develop new components. We may encounter some key informants or providers who are unwilling to participate. We expect this is unlikely given our success in recruiting practitioners for the published preliminary studies and for the ongoing work involving other stakeholders. In the unlikely event that study site participation is poor, we can identify additional sites of similar size, region, and affiliation. We will incentivize participation by providing a nonconditional cash incentive to interviewees and survey respondents. Finally, every setting is unique and not all successful practices can be adapted to other settings (eg, the organization’s support for implementation may vary). To optimize our feasibility study, we will engage stakeholders at the study sites in selecting tools and strategies from the toolkit. Identifying microlevel strategies and tools (ie, those that are essential in specific settings or populations) is a critical area for future work.

## Appendix

Multimedia Appendix 1 Peer reviews from the Agency for Healthcare Research and Quality.

## Abbreviations

AHRQ: Agency for Healthcare Research and Quality
CANDOR: Communication and Optimal Resolution
CFIR: Consolidated Framework for Implementation Research
ICANDOR: Intersystem Communication and Optimal Resolution
IMED: Intersystem Medical Error Discovery
NCI: National Cancer Institute
TDF: Theoretical Domains Framework
