# Supplementary material for: A Modified Communication and Optimal Resolution Program for Intersystem Medical Error Discovery: Protocol for an Implementation Study
Source: JMIR Res Protoc. 2019 Jul 2;8(7):e13396. doi: 10.2196/13396 (PMC6632107; doi:10.2196/13396)
Supplement: Multimedia Appendix 1 [file resprot_v8i7e13396_app1.pdf]

SUMMARY STATEMENT
(Privileged Communication)

PROGRAM CONTACT:
Tamara Willis
301-427-1011
tamara.willis@ahrq.hhs.gov

Release Date: **07/02/2018**
Revised Date:

Application Number: **1 K08 HS026030-01A1**

Principal Investigator

DOSSETT, LESLY A

Applicant Organization: UNIVERSITY OF MICHIGAN AT ANN ARBOR

Review Group: **HCRT**
HSR Health Care Research Training SS

Meeting Date: **05/24/2018**
Council: **OCT 2018**
Requested Start: **09/01/2018**
RFA/PA: **PA17-232**
PCC: **OEREP**

Project Title: **Error Identification Between Health Systems—A Patient Safety Blind Spot**

SRG Action: **Impact Score:21 Percentile:9**

Human Subjects: **48-At time of award, restrictions will apply**
Animal Subjects: **10-No live vertebrate animals involved for competing appl.**
Gender: **1A-Both genders, scientifically acceptable**
Minority: **1A-Minorities and non-minorities, scientifically acceptable**
Clinical Research - not NIH-defined Phase III Trial

Project Year	Direct Costs Requested	Estimated Total Cost
1	143,800	155,304
2	143,800	155,304
3	143,800	155,304
4	143,800	155,304
TOTAL	575,200	621,216

ADMINISTRATIVE BUDGET NOTE: The budget shown is the requested budget and has not been adjusted to reflect any recommendations made by reviewers. If an award is planned, the costs will be calculated by Institute grants management staff based on the recommendations outlined below in the COMMITTEE BUDGET RECOMMENDATIONS section.

1K08HS026030-01A1 Dossett, Lesly A

PROTECTION OF HUMAN SUBJECTS UNACCEPTABLE

RESUME AND SUMMARY OF DISCUSSION: This K08 grant application resubmitted by Dr. Lesly Dossett from the University of Michigan, and entitled, "Error Identification between Health Systems—A Patient Safety Blind Spot" responds to PA17-232, AHRQ Mentored Clinical Scientist Research Career Development Award (K08). The applicant proposes to assess potential strategies to facilitate transparent communication and optimal resolution of errors discovered between facilities. It will also evaluate whether these strategies are acceptable and feasible for implementation. The results will have immediate impact improving patient safety and improving cancer care quality. The applicant is a naval surgeon with unique perspective in health care research. Her mentors have outstanding track records of mentoring. Her history of research on cancer outcomes, health care delivery, and provider performance offer a good foundation for her planned research. The applicant and her research advisors have expertise in outcomes research, mixed methods research, implementation science, and bioethics. The environment at Michigan is ideal for promoting the careers of K researchers. Her research includes quantitative and qualitative methods, as well as a plan to pilot an intervention. Her work is novel and important. Her plan overall is well structured and thoughtful. The review committee believes physician communication across organizations is important and a significant subject for study. She will build on her quantitative expertise to develop skills in implementation science, through formal coursework, participation in targeted training programs and formal mentoring. The applicant has provided an elaborated training and research plan and application has a clear monitoring and evaluation plan in place. Approach adapts a well-known CANDOR process with stakeholder input to understand barriers and facilitators to its use based on CFIR in preparation for developing and testing an intervention. The research plan has been substantially reconsidered and the candidate is responsive to previous reviewers' comments. However, the reviewers are not clear how "errors" are defined, what the preliminary thoughts are on how an inter-hospital response teams works and the success in the research project rests heavily on I- CANDOR success. There is no comment on the effect on patients when a mistake is recognized. The applications approach lacks detail on the sample for the survey and quantitative analysis and does not directly address the bias introduced by Michigan's special environment. The application appears too ambitious for the time frame. Overall, the reviewers rated this application within "Outstanding" to "Exceptional" range with level of "High Impact."

DESCRIPTION (provided by applicant): Preventable medical errors represent a major public health problem. To prevent future errors, improve disclosure, and mitigate malpractice risks, organizations have adopted strategies for early transparent communication and emphasized quality improvement through peer review. These principles are incorporated into the AHRQ Communication and Optimal Resolution (CANDOR) Toolkit, which facilitates 1) transparent communication, 2) error prevention, and 3) achieving optimal resolution with patients and families. What physician's should do when they discover another physician's error is less clear. Application of the CANDOR process may be particularly complex when the discovering and responsible physicians practice in different facilities. Without clear expectations or mechanisms for disclosure and feedback on the part of the discovering provider, it is uncertain to what extent and in what way these errors are being communicated with patients or responsible providers. Further, known barriers to disclosure and reporting one's own error may not be relevant or replaced by other unknown barriers when considering transparent communication regarding another physician's error. Providing a communication and resolution strategy applicable to errors discovered between physicians and facilities would help address this current blind spot in the patient safety and quality improvement movement. We will investigate these questions through a series of studies engaging stakeholders. This research will be the first to develop strategies to improve physician-to-physician communication over sensitive patient scenarios representing complex ethical and professional challenges for many physicians. Furthermore, the research project, multidisciplinary mentorship team, and unparalleled research environment are ideally suited to address the career goals and educational needs of the candidate, Lesly Dossett, MD, MPH. The proposal includes a detailed

educational plan with training that will be essential for successful completion of this research and for Dr. Dossett's career development. The training includes mentored research and graduate level courses in implementation science. This career development award will lay the groundwork for Dr. Dossett to perform ongoing, innovative health services research, and to become an independent investigator.

PUBLIC HEALTH RELEVANCE: This project will assess potential strategies to facilitate transparent communication and optimal resolution of errors discovered between facilities. It will also evaluate whether these strategies are acceptable and feasible for implementation. The results will have immediate impact improving patient safety and improving cancer care quality.

CRITIQUES: The written critiques of individual reviewers are provided in essentially unedited form below. These critiques were prepared prior to the meeting and may not have been revised afterwards. The “Resume and Summary of Discussion” above summarizes the final opinions of the committee.

CRITIQUE 1

Candidate:	1
Career Development Plan/Career Goals/Plan to Provide Mentoring:	2
Research Plan	3
Mentor(s), Co-Mentor(s):	2
Environment Commitment to the Candidate	1

Overall Impact:
Strengths
- This uniquely qualified, well trained surgeon-scientist has a stellar mentoring team with a well elaborated training and research plan. Her background as a naval surgeon informs her career and research goals in important and productive ways. Her mentors are hands on with outstanding track records of mentoring. Her history of research on cancer outcomes, health care delivery, and provider performance offer a good foundation for her planned research. The question of how physicians communicate across organizations when a clinical mistake has been made is important and not well studied. Her research includes quantitative and qualitative methods, as well as a plan to pilot an intervention. Recognizing the requirements of this work, she has assembled mentors and consultants with a unique set of skills (e.g. outcomes research, mixed methods research, implementation science, and bioethics). Her training across several and research aims to first understand barriers to physician-to-physician communication. Her work is novel and important. Her plan overall is well structured and thoughtful. The environment at Michigan is ideal for promoting the careers of K researchers.

Weaknesses
- Her approach lacks detail on the sample for the survey and quantitative analysis and does not directly address the bias introduced by Michigan’s special environment. These are really the only minor weaknesses and they are not out of line from what I’d expect from someone at this career stage, and the team shows other ample evidence of strong and helpful input throughout the application. So I down-weight these weaknesses. My only concern is that she may be too ambitious, though she has packed in an impressive set of experience and training already, so this is negligible.

1. Candidate:
Strengths
- Candidate’s background as a naval surgeon offers unique perspective especially around managing risk, the topic of her training and research.
- Her clinical training, prior analytic work, and research prepared her well to pursue work that reduces risk.

- She has assembled a strong team of mentors, which speaks to the promise other seasoned researchers see in her.
- She already shows ample evidence of collaboration with her Michigan mentors even though she only arrived in 2016.
- Her research and training plan (as a whole) fit together very well.
- Since first submission, applicant and team published "Barriers to Disclosure and Feedback of Errors between Facilities," in July 2017 *Annals of Surgery*. Based on qualitative analysis of 30 semi-structured interviews with cancer specialists.

Weaknesses
- None noted.

2. Career Development Plan/Career Goals and Objectives:
Strengths
- Developing mixed methods and implementation science skills is a good fit for her goal of "moving evidence into practice."
- Good access to appropriate coursework on implementation science (2 formal courses) and qualitative mixed methods (2 formal courses and learning lab).
- Good path to R01 with R01 boot camp.

Weaknesses
- Too broad across several complex areas: primary data collection via surveys, qualitative data collection, and implementation science each could be their own focus of a K alone.

3. Research Plan:
Strengths
- Clear and strong scientific motivation for the project – errors in cancer care (as all medical care) are common yet there is no way to handle such errors when they cross facilities. The training and research aims all work towards addressing this need.
- By asking clear questions (i.e. what are barriers to communication of other physicians' errors between facilities?), the PI is likely to elicit useful and actionable information. She has greatly clarified the plan for each piece of her research aims.
- To modify the CANDOR process for use across facilities – applicant proposes highly structured plan combining evidence synthesis, independent review by stakeholder panel, face-to-face meetings of stakeholder panel, and summary of final best practice recommendation (Aim 1).
- 5 NCI-designated cancer centers targeted for 20 semi-structured interviews and 250 cancer specialist survey respondents (50 per site) to understand how inter facility CANDOR barriers/implementation differ from within facility CANDOR.
- 2 pilot sites, Michigan and Moffit where PI and team have deep connections, and therefore high likelihood of success with proposed research.
- Aim 3 more narrowly focused around implementation outcomes of feasibility and acceptability with more clearly detailed design. Use of toolkit (aim 2) and input from stakeholders to implement at 2 sites and measure well defined implementation outcomes including adoption (month 4-6), appropriateness, reach, acceptability, and feasibility (month 12).

Weaknesses
- The work proposed remains ambitious and any unanticipated delays outside the PI's control will make it impossible to complete the proposed work as each aim flows from the previous aim(s). This is a minor to moderate weakness balanced by the significance of the work, strength of the candidate and team.

4. Mentor(s), Co-Mentor(s), Consultant(s), Collaborator(s):
Strengths
- Exceptionally strong primary mentor (Dr. Dimick), himself a surgeon-scientist, with ample NIH funding and with five past K mentees,

- Clear mentoring goals like 1. Develop long term research agenda 2. Ensure completion of educational plan. 3. Help with dissemination of findings. 4. Facilitate transition to R01 research.
- Mentoring team nicely fits every aspect of training goals, with experts in mixed methods (Dr. Fetters), implementation science (Dr. Sales), and survey research (Jagsi).
- Space in close proximity (Dr. Jagsi) facilitates formal and informal meetings.
- Mentoring roles are clearly laid out.
- Very strong team of co-mentors with mixed-methods experience, and one with implementation science, and a consultant who developed "The Michigan Model" of error disclosure and health system claims.

Weaknesses

- Only some of the mentor letters were updated. Notably, Dimick (primary mentor) and Mulholland, chair of surgery, were not.

5. Environment and Institutional Commitment to the Candidate:
Strengths

- University of Michigan offers an outstanding environment of support and many research colleagues that has produced successful K recipients in the past.
- Dr. Dimick is guaranteeing access to CHOP's personnel, data management infrastructure, analysts, and computing systems.
- Her chair in Dept. of Surgery pledges 75% protected time and $180k in startup funds to help carry out the work.

Weaknesses

- None noted.

Training in the Responsible Conduct of Research:
Strengths

- While in the US Navy, PI was Special Assistant to the Commanding Officer for Research (overseeing8-10 human subject protocols).
- In addition to web based training, she has in person training planned. She will take Research Responsibility and Ethics (including 8 hours of faculty-facilitated small-group discussions across varied topics. (Fraud, data storage, peer review, animals, human subjects research, conflicts of interest, global workspace, dual use research.
- This training will be supervised by Dr. Dimick.

Weaknesses

- None noted.

Degree of Responsiveness:
Strengths

- The investigator clearly fits the goals of the K08 as an early stage investigator who needs additional skills and mentoring to achieve her career goals around mixed methods and implementation science work to improve physician to physician communication regarding errors.

Weaknesses

- None noted.

Inclusion of AHRQ Priority Populations:
Strengths

- Acceptable – the ultimate target of the work, oncology patients, span priority populations, but the immediate target, is their care providers.

Weaknesses

- None noted.

Budget and Period of Support:

Strengths

- Generally appropriate with minor concern below.

Weaknesses

- The primary data collection plus focus groups and coding seems like it could require more resources than available under a K, but could be supplemented (with her startup funds of $180k).

Inclusion of Women and Minority Subjects:
Strengths

- Acceptable – the ultimate target of the work, oncology patients, span both sexes and minorities, but the immediate target, is their care providers.

Weaknesses

- None noted.

Resubmissions:
Strengths

- The applicant is highly responsive to reviewer critiques yielding a clearer, streamlined plan for training and research. Specifically
- In response to diffuse and over-ambitious training goals, she has narrowed the scope to focus on stakeholder analysis, development of an implementation strategy, and implementation outcome assessment. She has completed 3-day training in mixed methods.
- To address lack of generalizability (Michigan is a unique setting with outsized attention to surgical quality), applicant proposes additional NCI-designated regional referral cancer centers to purposively sample 5 of 69 NCI-designated centers in Aim 2, and added Moffit Cancer Center in Tampa, FL for aim 3.
- To address the lack of details regarding sample frame, target survey respondents, recruitment strategy and content in Aim 2, applicant proposes targeting 50 cancer specialists from 5 sites (250) with a target RR of 65% (163), in line with successful recruitment by her mentors.
- To address need for specific implementation outcomes, applicant proposes primary outcomes of acceptability and feasibility and secondary outcomes of appropriateness, reach and adoption. Applicant will use dissemination and implementation Toolkit from Aim 2 to implement I-CANDOR at 2 study sites over 12 months.

Weaknesses

- None noted.

CRITIQUE 2

Candidate:	1
Career Development Plan/Career Goals/Plan to Provide Mentoring:	2
Research Plan	3
Mentor(s), Co-Mentor(s):	1
Environment Commitment to the Candidate	1

Overall Impact:
Strengths

- Very strong candidate with a track record for research and publication in areas relevant to patient safety – glowing letters from accomplished mentor team highlight her strengths and potential to become an independent researcher. Mentor team has exceptional track record for research and mentoring, and have complementary expertise relevant to the career development plan. She will build on her quantitative expertise to develop skills in implementation science, through formal coursework, participation in targeted training programs and formal mentoring. A clear monitoring and evaluation plan is provided. Environment is outstanding and supportive.

- Innovative question, as a framework for reporting between-institution errors isn't available. Approach adapts a well-known CANDOR process with stakeholder input to understand barriers and facilitators to its use based on CFIR, in preparation for developing and testing an intervention.

Weaknesses

- The proposal depends highly on the ability to apply CANDOR, which helps address provider and local systems factors when an error occurs, as a model for addressing errors recognized by others, from other institutions, in hindsight. I miss an analysis of the accuracy with which some errors can be identified given limited information on decision-making.

1. Candidate:

Strengths

- Surgical oncologist with clinical practice experience, focus on patient safety and communication between providers. Obtained her MD and surgical residency at Vanderbilt, then AHRQ T32 training and an MPH with quantitative training, and pursued a surgical oncology fellowship after 4 years of military service.
- Strong candidate with expertise in statistical modeling and large database analysis as applied to studies of practice variation and provider performance, clinical outcomes re: surgery for cancer; well published to date. Since the initial application, she also participated in a 3-day participatory mixed methods workshop.
- Strong letters of support from her mentor team attest to an extraordinarily promising candidate for a health services research career.

Weaknesses

- None noted

2. Career Development Plan/Career Goals and Objectives:

Strengths

- Current plan builds on quantitative expertise and preliminary study of PCP/cancer specialist communication to build formal training in implementation science to study error communication between providers at different institutions, with goal of developing and piloting an intervention that can be formally tested through an R01 in the future.
- The plan includes formal coursework in implementation science (2 courses), targeted readings and seminars. Plans for monitoring and evaluation are presented with weekly meetings with Dr. Dimick, twice monthly with Dr. Jagsi, and monthly with other mentors – all will meet biannually for feedback, concerns; annual modification of timeline and action steps.
- Project is of priority to AHRQ, as builds on the CANDOR model to improve communication about errors with a goal to improve patient safety.

Weaknesses

- None noted.

3. Research Strategy:

Strengths

- Problem statement and rationale suggest need for inter-facility identification and communication re: medical errors, especially in the complex care of cancer patients, and current lack of professional guidelines on this topic.
- The topic is highly relevant to her previous work and career goals.
- Her aims include 1) to develop a guideline for best practice re: transparent communication and resolution of errors between facilities using an expert panel process; 2) conduct a mixed methods study with stakeholders to understand barriers and facilitators to achieving the best practice from Aim 1; and 3) Develop and pilot an intervention adapted from the CANDOR toolkit.
- Conceptual models to be used are clear, and methods for Aims 1 and 2a are well described and appropriate where possible.

- Aim 2 was expanded to include additional NCI designated cancer centers, in addition to UMich, and greater details for the survey administration provided – this seems clear.
- Though content of the intervention is pending results from Aims 1 and 2, the application now includes the framework for evaluating the outcomes of the intervention.

Weaknesses

- I have questions re: the ability of providers to accurately assess (at least some) errors of others given limited information on context and decision-making, and of the application of CANDOR, which helps address provider and local systems factors when an error occurs, as a model for addressing errors recognized by others, from other institutions, in hindsight.

4. Mentor(s), Consultant(s), Collaborator(s):
Strengths
Strong mentorship team with complementary expertise and mentoring track records:

- Dimick: Professor of Surgery and Director of the Center for Healthcare Outcomes and Policy at U Michigan.
- Jagsi – Radiation Oncology and Dir Center for Bioethics and Social Sciences in Medicine – qualitative and quantitative social science research, mixed methods, gender equity and career development.
- Sales: Prof of Nursing, Implementation science
- Fetters: Prof Fam Med – Mixed methods research
- Boothman (consultant) JD – exec. Dir. of clinical safety and chief risk officer.
- Mentors are highly productive with multiple publications and grant activity; and provide strong letters of support attesting to the candidate's potential and the institution's commitment.

Weaknesses

- None noted.

5. Environment and Institutional Commitment to the Candidate:
Strengths

- Excellent research environment
- Strong letter from Surgery Chair committing to 75% protected time, including provision of startup funds, space and institutional support.

Weaknesses

- None noted

Degree of Responsiveness:
Strengths

- Highly responsive, focusing on communication re: medical errors between institutions, and preparation for a career in HSR.

Weaknesses

- None noted.

Budget and Period of Support:
Strengths

- Seems appropriate

Weaknesses

- None noted.

Inclusion of Women and Minority Subjects: Acceptable

Inclusion of AHRQ Priority Populations: Acceptable

Protection of Human Subjects from Research Risks: Acceptable

Privacy and Security Protections for Patients: None noted.

Resubmission Applications (formerly "revised/amended" applications):
Strengths
- The resubmission is responsive to reviewer comments. She has narrowed the scope of training to focus on implementation science, as there was concern that combining with mixed methods training was overly ambitious, and she has obtained some additional mixed methods training in the meantime. Additional NCE-designated cancer centers were added to address concern of the unique nature of the UMich site, and further developed the methodologies for the survey proposed in Aim 2, and the Framework for evaluating outcomes of Aim 3.

Weaknesses
- None noted.

CRITIQUE 3

Candidate:	1
Career Development Plan/Career Goals/Plan to Provide Mentoring:	2
Research Plan	3
Mentor(s), Co-Mentor(s):	1
Environment Commitment to the Candidate	1

Overall Impact:
Strengths
- The research plan has been substantially re-considered and the candidate was responsive to reviewer comments
- Great candidate who will likely be successful.

Weaknesses
- Success in the research project rests heavily on I-Candor success.
- There is still a fair amount left to imagination—how "errors" are defined, what the preliminary thoughts are on how an inter-hospital response teams works.
- Philosophically, it is not clear what the impact of an error-reporting system is in the absence of other communication mechanisms intended to facilitate dissemination of knowledge and best practice. This seems rooted in a perceived notion of a two-tiered system of those who are knowledgeable and those who are not, rather than one in which a referral facility extends its reach to improve knowledge of cancer care system wide. Who is the "judge" of whether there is a difference in opinion or difference in practice
- There is no comment on the effect on patients---care should be taken to ensure this is managed. For example, for CANDOR, when a mistake is recognized, there is a framework for *immediate* disclosure. When one MD/health system blames another, they may be right or wrong. How is the impact of this managed?

1. Candidate:
Strengths
- Has demonstrated a commitment to discovery as evidenced by time spent acquiring research skills, and through continued publication despite an 8-year break to complete clinical obligations between research years and beginning a faculty job.

Weaknesses
- None noted.

2. Career Development Plan/Career Goals and Objectives:
Strengths
- Revisions now include additional training and a clearer development plan.

Weaknesses

- None noted

3. Research Plan:

Strengths

- The aims deploy validated tools in novel ways to sequentially build towards a communications framework.

Weaknesses

- Success in the research project rests heavily on I-Candor success.
- There is still a fair amount left to imagination—how "errors" are defined, what the preliminary thoughts are on how an inter-hospital response teams works.
- Philosophically, it is not clear what the impact of an error-reporting system is in the absence of other communication mechanisms intended to facilitate dissemination of knowledge and best practice. This seems rooted in a perceived notion of a two-tiered system of those who are knowledgeable and those who are not, rather than one in which a referral facility extends its reach to improve knowledge of cancer care system wide. Who is the "judge" of whether there is a difference in opinion or difference in practice
- There is no comment on the effect on patients---care should be taken to ensure this is managed. For example, for CANDOR, when a mistake is recognized, there is a framework for *immediate* disclosure. When one MD/health system blames another, they may be right or wrong. How is the impact of this managed?

4. Mentor(s), Co-Mentor(s), Consultant(s), Collaborator(s):

Strengths

- Mentor with proven track record.

Weaknesses

- None noted.

5. Environment and Institutional Commitment to the Candidate:

Strengths

- Excellent environment.

Weaknesses

- None noted.

Training in the Responsible Conduct of Research: Acceptable

Degree of Responsiveness: Acceptable

Inclusion of AHRQ Priority Populations: Acceptable

Budget and Period of Support: Acceptable

Inclusion of Women and Minority Subjects: Acceptable

Privacy and Security Protections for Patients: Acceptable

Resubmissions:

Strengths

- Added sufficient detail to research plan

Weaknesses

- Did not address many key concerns

THE FOLLOWING SECTIONS WERE PREPARED BY THE SCIENTIFIC REVIEW OFFICER TO SUMMARIZE THE OUTCOME OF DISCUSSIONS OF THE REVIEW COMMITTEE, OR REVIEWERS' WRITTEN CRITIQUES, ON THE FOLLOWING ISSUES:

PROTECTION OF HUMAN SUBJECTS: UNACCEPTABLE
The information on the protection of human subjects from research risks is inadequate to determine if there is adequate protection against risks. The applicant should recognize and be prepared to deal with effect of mistake discovery on patients.

INCLUSION OF WOMEN PLAN: ACCEPTABLE

INCLUSION OF MINORITIES PLAN: ACCEPTABLE

INCLUSION OF CHILDREN PLAN: ACCEPTABLE

COMMITTEE BUDGET RECOMMENDATIONS: The budget was recommended as requested.

MEETING ROSTER

HSR Health Care Research Training SS
Health Services Research Initial Review Group
AGENCY FOR HEALTHCARE RESEARCH AND QUALITY
HCRT
05/24/2018 - 05/25/2018

CHAIRPERSON(S)

HSU, JOHN, MD, MBA, MSCE
ASSOCIATE PROFESSOR OF MEDICINE
PROGRAM FOR CLINICAL ECONOMICS AND POLICY ANALYST
MONGAN INSTITUTE FOR HEALTH POLICY
MASSACHUSETTS GENERAL HOSPITAL
BOSTON, MA 02114

MEMBERS

ANCKER, JESSICA S., MPH, PHD
ASSOCIATE PROFESSOR
DEPARTMENT OF HEALTHCARE POLICY AND RESEARCH
WEILL CORNELL MEDICAL COLLEGE
NEW YORK, NY 10065

ARBAJE, ALICIA INES, MD, MPH, PHD
ASSOCIATE PROFESSOR OF MEDICINE,
ASSISTANT PROFESSOR OF MEDICINE
JOHNS HOPKINS CTR FOR TRANSFORM. GERIATRICS RES
JOHNS HOPKINS UNIVERSITY SCHOOL OF MEDICINE
5200 EASTERN AVE. MASON F. LORD 711
BALTIMORE, MD 21224

BURKHART, ELIZABETH, PHD
ASSOCIATE PROFESSOR
MARCELLA NIEHOFF SCHOOL OF NURSING
LOYOLA UNIVERSITY CHICAGO
CHICAGO , IL 60626

BURNS, EDITH A., MD
PROFESSOR OF MEDICINE
DIISION OF GERIATRICS
MEDICAL COLLEGE OF WISCONSIN
CLINICAL PROFESSOR OF MEDICINE
MILWAUKEE, WI 53295

DESHPANDE, ABHISHEK, MBBS, PHD *
ASSISTANT PROFESSOR
DEPARTMENT OF MEDICINE
CLEVELAND CLINIC LERNER COLLEGE OF MEDICINE
CASE WESTERN RESERVE UNIVERSITY
CLEVELAND, OH 44106

ELDER, NANCY C., MD, MPH
PROFESSOR
DEPARTMENT OF FAMILY AND COMMUNITY MEDICINE
UNIVERSITY OF CINCINNATI
CINCINNATI, OH 45236

FREUND, KAREN, MD
PROFESSOR AND VICE CHAIR OF MEDICINE
INSTITUTE FOR CLINICAL RESEARCH AND HEALTH POLICY STUDIES TUFTS MEDICAL CENTER
TUFTS UNIVERSITY
BOSTON, MA 02111

FUJI, KEVIN T., PHMD
ASSOCIATE PROFESSOR AND DIRECTOR
CENTER FOR HEALTH SERVICES RESEARCH & PATIENT SAFETY
CREIGHTON UNIVERSITY
OMAHA, NE 68178

HALL, ALLYSON GAIL JR, PHD *
PROFESSOR
MANAGEMENT AND POLICY
UNIVERSITY OF ALABAMA AT IRMINGHAM
BIRMINGHAM, AL 35212

HAWK, MARY ELIZABETH, DRPH *
ASSISTANT PROFESSOR
OFFICE OF RESEARCH
UNIVERSITY OF PITTSBURGH
PITTSBURGH, PA 15213

IRWIN, CHARLES E., MD
DISTINGUISHED PROFESSOR OF PEDIATRICS
DIRECTOR, DIVISION OF ADOLESCENT & YOUNG ADULT MED
DIRECTOR, HEALTH POLICY, DEPT. OF PEDIATRICS
UCSF BENIOFF CHILDREN'S HOSPITAL
UNIVERSITY OF CALIFORNIA, SAN FRANCISCO
SAN FRANCISCO, CA 94143--050

KROTH, PHILIP J., MD *
PROFESSOR
HEALTH SCIENCES LIBRARY AND INFORMATICS CENTER
UNIVERSITY OF NEW MEXICO
ALBUQUERQUE, NM 87131

LITVIN, CARA B., MD, MS
ASSOCIATE PROFESSOR
DIVISION OF GENERAL INTERNAL MEDICINE & GERIATRIC
DEPARTMENT OF MEDICINE
MEDICAL UNIVERSITY OF SOUTH CAROLINA
CHARLESTON, SC 29425

LOVE, THOMAS EZRA, PHD *
PROFESSOR OF MEDICINE, EPIDEMIOLOGY AND BIOSTATISTICS
DIRECTOR, BIOSTATISTICS AND EVALUATION UNIT
CENTER FOR HEALTH CARE RESEARCH AND POLICY
CASE WESTERN RESERVE UNIVERSITY
CLEVELAND , OH 44109

MAYS, GLEN P., PHD, MPH
PROFESSOR AND CHAIR
HEALTH SERVICES AND SYSTEMS RESEARCH
COLLEGE OF PUBLIC HEALTH
UNIVERSITY OF KENTUCKY
LEXINGTON, KY 40536

MCHUGH, MEGAN, PHD
ASSISTANT PROFESSOR OF EMERGENCY MEDICINE
NORTHWESTERN UNIVERSITY
FEINBERG SCHOOL OF MEDICINE
CHICAGO, IL 60611

MEARA, ELLEN R., PHD
ASSOCIATE PROFESSOR
DARTMOUTH INSTITUTE FOR HEALTH POLICY
AND CLINICAL PRACTICE
GEISEL SCHOOL OF MEDICINE AT DARTMOUTH
LEBANON, NH 03766

MERWIN, ELIZABETH I., PHD
ANN HENSHAW GARDINER PROFESSOR OF NURSING
EXECUTIVE VICE DEAN
DUKE UNIVERSITY SCHOOL OF NURSING
SCHOOL OF MEDICINE
DEPARTMENT OF PSYCHIATRY &BEHAVIOR SCIENCE
DURHAM, NC 27710

MEURER, LINDA N., MPH, MD *
PROFESSOR
FAMILY AND COMMUNITY MEDICINE
MEDICAL COLLEGE OF WISCONSIN
MILWAUKEE, WI 53226

MULLINS, C. DANIEL, PHD, MA
PROFESSOR
DEPARTMENT OF PHARMACEUTICAL HEALTH
SERVICES RESEARCH
UNIVERSITY OF MARYLAND SCHOOL OF PHARMACY
BALTIMORE, MD 21201

NAIK, AANAND D., MD
ASSOCIATE PROFESSOR OF MEDICINE AND VICE CHAIR
HOUSTON CENTER FOR INNOVATIONS IN QUALITY,
SAFETY, AND EFFECTIVENESS
DEPARTMENT OF MEDICINE
BAYLOR COLLEGE OF MEDICINE
HOUSTON, TX 77004

NEWGARD, CRAIG D.,MD, MPH
PROFESSOR OF EMERGENCY MEDICINE
DEPARTMENTS OF EMERGENCY MEDICINE
AND PUBLIC HEALTH
OREGON HEALTH AND SCIENCE UNIVERSITY
PORTLAND, OR 97239

PETERSON, KEVIN ARTHUR, MD, MPH
PROFESSOR
DIRECTOR, CENTER OF EXCELLENCE IN PRIMARY CARE
DEPARTMENT OF FAMILY MEDICINE AND COMMUNITY HEALTH
UNIVERSITY OF MINNESOTA
MINNEAPOLIS, MN 55105

SCHMID, CHRISTOPHER H., PHD
PROFESSOR OF BIOSTATISTICS
CENTER FOR EVIDENCE SYNTHESIS IN HEALTH
BROWN UNIVERSITY SCHOOL OF PUBLIC HEALTH
PROVIDENCE, RI 02912

SCOTT, LINDA D., PHD
DEAN, & PROFESSOR
SCHOOL OF MEDICINE
UNIVERSITY OF WISCONSIN MADISON
MADISON, WI 53705

STAUDENMAYER, KRISTAN L., MD, MS, FACS, MD *
ASSOCIATE PROFESSOR OF SURGERY GORDON AND
BETTY MOORE ENDOWED FACULTY SCHOLAR
DIVISION OF TRAMA EMERGANCY SURGERY
AND SURGICAL CRITICAL CARE
DEPARTMENT OF SURGERY
STANFORD UNIVERSITY MEDICAL CENTER
STANFORD, CA 94305

SUMNER, WALTON, MD
ASSOCIATE PROFESSOR
DEPARTMENT OF INTERNAL MEDICINE
WASHINGTON UNIVERSITY SCHOOL OF MEDICINE
ST. LOUIS, MO 63110

WISNIVESKY, JUAN P., MD
PROFESSOR OF MEDICINE
DEPARTMENT OF MEDICINE
MOUNT SINAI SCHOOL OF MEDICINE
NEW YORK, NY 10029

SCIENTIFIC REVIEW OFFICER

AZADEGAN, ALI A., PHD, DVM
SCIENTIFIC REVIEW OFFICER
DIVISION OF SCIENTIFIC REVIEW (DSR)
OFFICE OF EXTRAMURAL RESEARCH, EDUCATION
& PRIORITY POPULATIONS (OEREP)
AGENCY FOR HEALTHCARE RESEARCH & QUALITY (AHRQ)
ROCKVILLE, MD 20857

EXTRAMURAL SUPPORT ASSISTANT

ROBINSON, DIANE
PROGRAM ANALYST
DIVISION OF SCIENTIFIC REVIEW (DSR)
OFFICE OF EXTRAMURAL RESEARCH, EDUCATION
& PRIORITY POPULATIONS (OEREP)
AGENCY FOR HEALTHCARE RESEARCH & QUALITY (AHRQ)
ROCKVILLE, MD 20857

* Temporary Member. For grant applications, temporary members may participate in the entire meeting or may review only selected applications as needed.

Consultants are required to absent themselves from the room during the review of any application if their presence would constitute or appear to constitute a conflict of interest.
